# Ordering Enhancement of Ion Bombardment-Induced Nanoripple Patterns: A Review

**DOI:** 10.3390/nano15060438

**Published:** 2025-03-13

**Authors:** Ying Liu, Hengbo Li, Chongyu Wang, Gaoyuan Yang, Frank Frost, Yilin Hong

**Affiliations:** 1 National Synchrotron Radiation Laboratory, University of Science and Technology of China, Hezuohua South Road 42, Hefei 230029, China; lihengbo@mail.ustc.edu.cn (H.L.); sa24231065@mail.ustc.edu.cn (C.W.); ygyuan@mail.ustc.edu.cn (G.Y.); ylhong@ustc.edu.cn (Y.H.); 2 Leibniz Institute of Surface Engineering (IOM), Permoserstraße 15, 04318 Leipzig, Germany; frank.frost@iom-leipzig.de

**Keywords:** ion bombardment, self-organization, ripples, nanostructures, ordering, ripple superimposition, guided self-organization

## Abstract

Low-energy ion bombardment (IB) has emerged as a promising, maskless nanofabrication tool for quasi-periodic nanoripples, marked by a high throughput and low cost. As templates, these IB-induced, self-organized surface nanoripples have shown potential for applications in diverse fields. However, the challenge of tailoring the ordering of these ripple patterns is preventing the widespread application of IB. Moreover, the enhancement of the ordering of these self-organized nanostructures involves the fundamental academic questions of nanoripple coupling (or superimposition) and guided self-organization. This review first focuses on the experimental progress made in developing representative strategies for the ordering enhancement of IB-induced nanoripples in terms of ion beams and targets. Second, we present our understanding of these developments from the perspectives of ripple superposition and guided self-organization. In particular, the basic conditions for ripple superposition under the non-conservation of mass are deduced based on the common features of the results from rocking bombardments of a single material and the bombardment of bilayer systems, providing insight into the mechanisms at play and deepening our understanding of these experimental observations. Finally, areas for future research are given, with the aim of improving ripple ordering from the viewpoints of ripple superimposition and guided self-organization. All this may re-stimulate interest in this field and will be of importance in advancing the academic research and practical applications of IB-induced nanopatterns.

## 1. Introduction

Ion bombardment (IB), which is based on a broad [1,2,3,4,5,6,7,8] or focused [9,10,11,12,13] ion beam, can induce self-organized nanoripples on the surface of various solid materials without a mask and with high throughput. Recently, as unique templates, IB-induced nanoripple structures have been used in fields such as surface plasmonics [14,15,16,17], optoelectronics [18,19], photovoltaics [20,21], flexible electronics [22], photocatalysis [23], magnetism [24,25], biomaterials [26,27], and wettability [28,29,30]. In addition, IB-induced nanostructures including nanoripples have the potential to be used in quantum nanoplasmonics [31,32]. Table S1 presents more detailed information on IB-induced nanoripples and the main bombardment conditions used for those with further interest in the matter (see Supplementary Materials).

The study of self-organized nanoripples induced by IB includes theoretical simulation [1,2,3,4,5,6,7,8], represented by the Bradley–Harper model [1], and experimental research on the basic evolution characteristics of the IB-induced nanoripples seen on different materials, including inorganic materials (e.g., metals, semiconductors, and insulators) and organic materials (e.g., polymers) [1,2,3,4,5,6,7,8,33,34,35]. As a promising nanofabrication tool, IB has attracted increasing attention. However, the study of the ordering of IB-induced nanoripples is relatively sparsed. Therefore, similar to other self-organized structures, improving the ordering of such nanoripples is a long-standing challenge in this field that involves fundamental academic issues such as ripple superimposition [36,37,38,39,40,41,42,43] and guiding strategies [44,45,46]. Furthermore, the low degree of ordering is also a technical bottleneck affecting the application of IB-induced nanoripples in industry.

In fact, optimizing IB parameters, especially the ion fluence [47,48], is important for obtaining nanoripples with a defect density that is as low as possible. However, this method of suppressing defects is limited. In other words, once the target material is determined, the range of ion fluence that is optimal for low-defect density ripples is limited. Thus, our ability to adjust the structural parameters of low-defect density ripples is also limited. In addition, efforts to improve the ordering of ripples have been made through several variations in the bombardment strategy: for a single material, a series of non-traditional bombardment methods have been developed for initially flat surfaces, such as sequential ion beam sputtering [49], moving [50], rocking [51,52], and intermittent bombardment [53]. Note that most of these studies were performed on Si surfaces. The reason for this lies in the fact that Si is an important material for technical applications and also a mono-elemental system, making it easier to understand IB-induced pattern formation mechanisms. Moreover, another study used a grating-prepatterned surface for bombardment to guide the growth of ripples [54]. In addition, bilayer systems have also been studied in terms of IB [55], with the same aim of enhancing ripple ordering. In our opinion, all these observations of the improvement in ripple ordering lay the cornerstone for exploring the best conditions for ripple superimposition [36,37,38,39,40,41,42,43] and developing new ideas for guiding ripple growth.

In this context, this study will review the experimental progress made in the ordering improvement (or defect suppression) of IB-induced nanoripples. Section 2 and Section 3 introduce the traditional and non-traditional IB of single materials, respectively. Section 4 details the bombardment of bilayer systems. Section 5 further analyzes and discusses the above research from the perspectives of ripple superimposition and guided self-organization. Finally, in Section 6, a summary of this study is provided, and an overview of the ordering enhancement of IB-induced nanoripples is offered from the perspective of practical applications.

## 2. Conventional Ion Bombardment of Single Materials

### 2.1. Conventional IB of Initially Flat Si Surfaces

**Figure 1:**
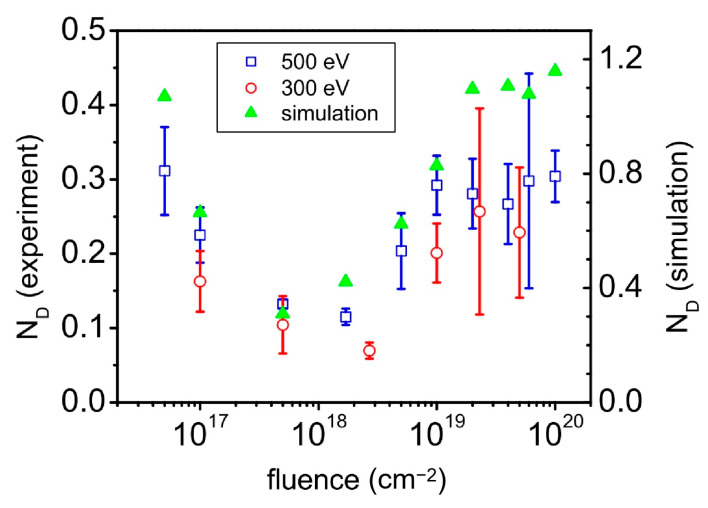
Normalized defect density of the experimental and simulated morphologies as a function of ion fluence [47]. Measured data are given at different ion energies [47].

Ion fluence plays an important role in tailoring the morphological parameters and ordering of nanostructures (e.g., nanoripples [47] and nanodots [48]) during IB. In particular, in 2008, A. Keller et al. studied the evolution of the normalized defect density of nanoripples produced on an initially flat Si (100) surface with increasing ion fluence under Ar-ion bombardment at an incident angle of 67° and different ion energies (Figure 1) [47].

Figure 1 shows the evolution of the normalized defect density (N_D_) as a function of ion fluence at different ion energies. The lower the N_D_, the better its ordering. As shown in Figure 1, both ion energy and ion fluence may affect the defect density. At a specific ion energy, the N_D_ changes from decreasing to increasing with increasing ion fluence (or bombardment time). In other words, the evolution of N_D_ with the ion fluence shows a similar trend at different ion energies. This can be understood from the effect of ion energy on the relaxation rate during IB [2]. Furthermore, with increasing ion fluence, the normalized defect density N_D_ evolves from decreasing to its minimum at an ion fluence of approximately 10^18^/cm^2^ and further increases with increasing ion fluence at each ion energy. Therefore, there is an optimized ion fluence or a small range of ion fluence that minimizes the N_D_ value. This means that with increasing fluence (or bombardment time), the IB-induced nanoripples evolve from development to degradation, corresponding to a decrease and increase in N_D_. In fact, this is a common issue of self-organization, including IB, in which defects appear pronounced at high fluence. In detail, in the development stage, the rippled nanostructures grow with improved ordering, corresponding to a decrease in N_D_. Subsequently, the minimum N_D_ value indicates the best ordering of the nanoripples, which also corresponds to the optimized ion fluence. Moreover, the ordering of nanoripples degrades with increasing fluence while defects, including interstitials and bifurcations, emerge. Thus, the ordering of the nanoripples worsens, and N_D_ increases significantly in this stage. In addition, the experimental results of this observation were consistent with the simulation results using the damped Kuramoto–Sivashinsky equation [56].

In this study, A. Keller et al. [47] extended the quantitative evaluation of defects or ordering of nanoripples from the power spectrum density (PSD) to the normalized defect density, which incorporates the morphological details of defects. Thus, the importance of ion fluence is emphasized when adjusting the ordering of the nanoripples during IB. On the other hand, it is worth noting that the optimized fluence for the minimum defect density is almost determined once the material and the bombardment facility are selected. This means that the capability of ion fluence to adjust the wavelength and amplitude of nanoripples is limited. Therefore, there is an urgent need to develop other methods to improve IB-induced nanoripples.

### 2.2. Conventional IB of Si with Periodic Prepatterns

In order to improve the ripple ordering, naturally, diffraction gratings with periodic structures fabricated by lithographic technologies can be considered to guide the growth of nanoripples during IB. This section reports representative research results on the bombardment of a prepatterned surface when nanoripples can be generated.

**Figure 2:**
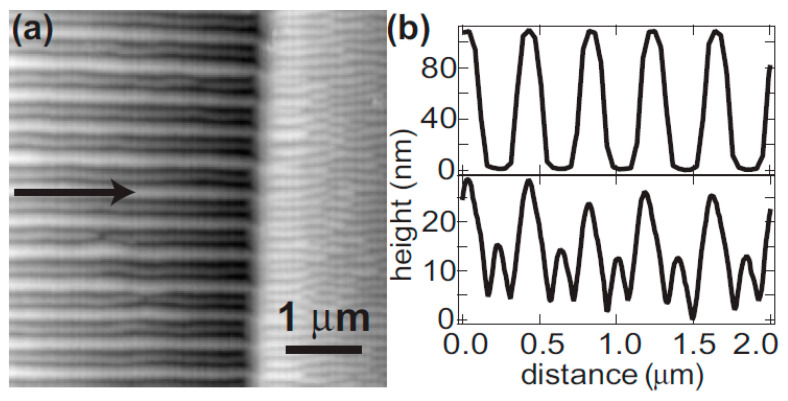
(**a**) Morphologies of the irradiated Si (001) surface with (left-hand side of image) and without (right-hand side of image) grating templates. Correspondingly, few (left-hand side of image) and many (right-hand side of image) morphological defects are visible on the irradiated Si (001) surface. The arrow in (**a**) indicates the direction of the projected ion beam. (**b**) Cross-section lines of AFM images across templated ridges before (top) and after (bottom) bombardment [54].

In 2005, A. Cuenat et al. studied the ordering of nanoripples by irradiating lateral grating templates fabricated using electron beam lithography (EBL) and focused ion beam (FIB) etching [54]. The period of grating templates is between 400 nm and 800 nm. Figure 2b shows a cross-sectional view of the morphology before and after the bombardment. Since the grating structures were bombarded under the condition that nanoripples can be induced, it is shown that nanoripples are visible in the valleys of the grating [lower plot in Figure 2b]. Moreover, guided by grating ridges, regular nanoripples developed near the edges of the grating ridges, which reduced defects, such as interstitials and bifurcations.

The study by A. Cuenat et al. [54] observed improved ripple ordering by the irradiation of prepatterns with grating structures, clearly demonstrating the guidance of periodic templates for self-organized nanoripples. In addition, for the first time, normalized defect density was proposed and used in this study to quantitatively evaluate defects.

This study is relevant to the issue of guided self-organization. In principle, prepattern structures can be fabricated using lithographic technologies such as EBL, FIB, and interference lithography. However, these lithographic technologies do not match IB in terms of either writing mode (EBL or FIB) or critical dimension (interference lithography). Hence, it is still challenging to apply the grating prepatterning strategy widely for the ordering enhancement of nanoripples.

## 3. Unconventional Ion Bombardment of Single Materials

### 3.1. Sequential IB

Sequential ion bombardment usually refers to sequentially bombarding the surface of a material at different conditions (ion beam incidence angle, energy, fluence, etc.). Usually, the first bombardment step is performed under the condition that nanoripples can be produced, whereas the second ion bombardment step plays an important role in the resulting morphology of the irradiated surface. For instance, various topographies, from nanobeads [40,42], the decay of initial ripples and growth of new ripples [41], to ordered nanoripples [49], can be observed, followed by the second IB step under different conditions. This section focuses on studies of nanoscale ripple patterns with improved ordering produced by sequential ion-beam sputtering [49].

**Figure 3:**
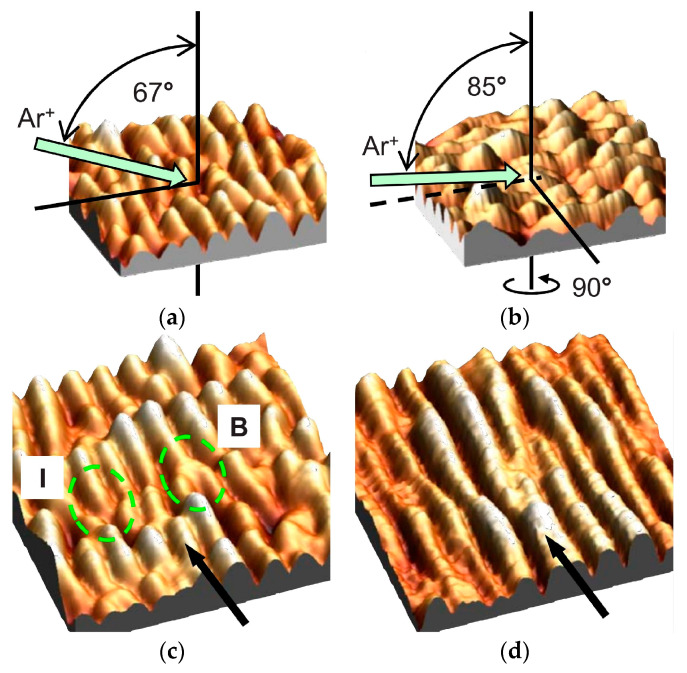
Schematic of the geometry of the sequential ion bombardment of a Si (100) surface after (**a**) the first and (**b**) the second bombardment steps. Three-dimensional plots in (**c**,**d**) show the irradiated Si (100) surface at a fluence of 6.9 × 10^15^ and 1.6 × 10^16^ cm^−2^, respectively [49]. The black arrows in (**c**,**d**) show the direction of the second ion beam sputtering. A bifurcation (B) and an interstitial (I) are indicated in (**c**). The left-handed and right-handed green circles show the defect areas of B and I, respectively.

In 2010, A. Keller and S. Facsko experimentally demonstrated the method to tune the quality of nanoripples through sequential ion bombardment, as shown in Figure 3. First, 500 eV Ar ions at an incidence angle of 67° were bombarded to form a conventional nanoripple morphology on the Si (100) surface, where the projection direction of the ion beam was parallel to that of the ripple vector. After the first bombardment step, the sample was rotated by an azimuthal angle of 90° and further irradiated with 500 eV Ar ions at an incidence angle of 85°. At grazing incidence, the pattern defects, i.e., the protruding parts of the nanoripples along the horizontal and vertical directions, tend to be preferentially etched. Hence, the uniformity of the ripple structure parameters was improved by reducing ripple defects, for example, an interstitial (I) and a bifurcation (B), as indicated in Figure 3c. In this study, the final normalized defect density (N_D_) of the ordered nanoripple pattern on a Si (100) surface with a period of 25 nm decreased from ~0.45 to ~0.26. In this study, the numerical integrations of the Kuramoto–Sivashinsky (KS) equation [49] on this sequential IB process can reproduce the above experimental observations, that is, the effect of the ordering improvement of the nanoripple patterns.

Note that in the second bombardment step, the projection direction of the ion beam is parallel to that of the ripple ridges, and no ripple pattern is formed on the irradiated surface. The essence of this process can be understood as the precise trimming of nanoripples owing to the preferential sputtering of pattern defects at grazing incidence.

### 3.2. Dynamic (Rocking) Bombardment of Si and Amorphous-Carbon

The regularity of the self-organized nanoripples can be enhanced by varying the relative position between the ion beam and the sample during IB. For example, the R. M. Bradley group theoretically proposed to improve the regularity of nanoripples through moving [50] and rocking [51,52] a substrate in 2012 and 2016, respectively. In this section, representative experimental progress on ordered nanoripples produced by rocking bombardment is shown [52].

**Figure 4:**
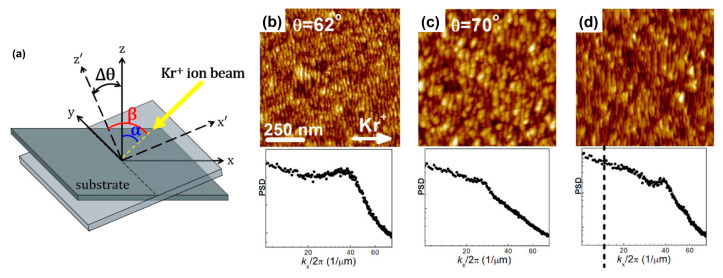
(**a**) Schematic diagram of the rocking geometry during IB. *θ* (e.g., equal to *α* and *β* in this sketch) is the polar angle between the incident ion beam (yellow arrow) and the surface normal of the unrocked substrate. The sample is rocked around the *y* axis, which is perpendicular to the projection of the beam on the (*x*,*y*) substrate plane. During rocking bombardment, *θ* varies periodically between the fixed values *α* and *β*, with the span of polar incidence angles Δ*θ* = *β* − *α*. AFM images of Si (100) surfaces at an incidence angle of (**b**) 62° and (**c**) 70° without rocking, and (**d**) with rocking, during which *θ* varies from 62° to 70°. This figure is reproduced from Figure 1 and Figure 3 in [52].

In 2020, Jo et al. experimentally demonstrated the ordering improvement of surface nanoripple patterns on Si and amorphous carbon targets with a rocking substrate under IB irradiation (Figure 4a). For comparison, Figure 4b,c show AFM images of the nanoripples formed on the Si surface at incidence angles of 62° and 70° without rocking during IB. As shown in Figure 4d, the nanoripples formed on the Si surface under rocking conditions exhibited better ordering than those without rocking. Note that the range of rocking frequencies varies from 0.017 min^−1^ to 0.275 min^−1^, which is wider than—and thus includes—the range for the optimal frequency as estimated by M. P. Harrison and R. M. Bradley [51]. Their experimental findings agree with the overall theoretical prediction based on the anisotropic KS equation proposed by M. P. Harrison and R. M. Bradley [51].

It is worth mentioning that at any incidence angle within the rocking range, nanoripple structures with similar lateral critical dimensions (i.e., wavelength) of nanoripples and ripple vectors in the same direction can be formed. In this case, the direction of the ripple vectors formed at any incidence angle was parallel to that of the projection of the incident ion beam on the sample surface.

Compared with previous research on dynamic bombardment, this study is unique in that a relatively narrow window of ion beam parameters has been found. In particular, during this rocking bombardment, the “constructive” growth between the existing and latent ripples can be maintained under non-conservation of mass by varying the incidence polar angle with an optimized frequency. This finding provides a practical research strategy for theoretical and experimental studies of ripple superposition.

## 4. Conventional Ion Bombardment of a Bilayer Material

**Figure 5:**
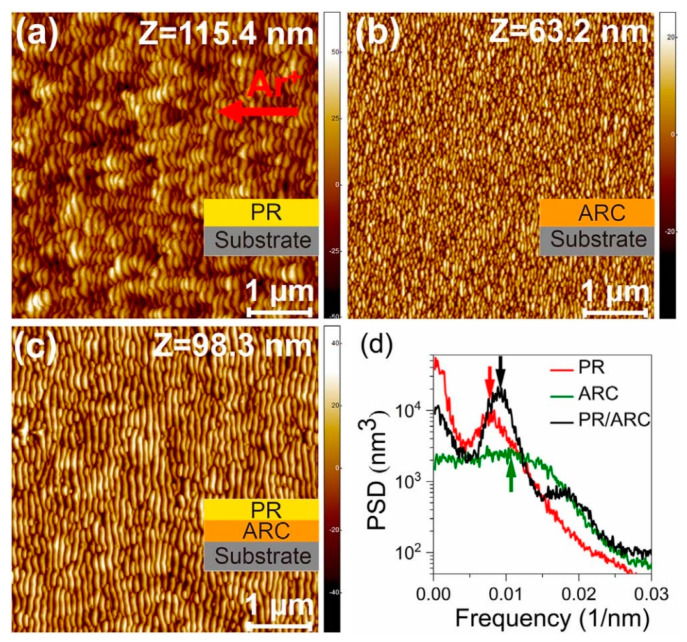
AFM images of the typical morphology on the (**a**) single PR, (**b**) single ARC, and (**c**) PR/ARC bilayer surfaces. (**d**) PSD curves of the AFM images shown in (**a**–**c**). The arrows show the ripple wavelengths for the three cases [55].

In addition to the studies on the ordering improvement of nanoripples on a single material in Section 2 and Section 3, the ordering improvement of nanoripples was also reported on a bilayer material, i.e., a bilayer system [55]. Figure 5a–c show AFM images of the nanoripples on the initially flat surfaces of a single photoresist (PR), single antireflection coating (ARC), and a PR/ARC bilayer system, respectively. The power spectral density (PSD) curve of each AFM image is shown in Figure 4d. All the samples were bombarded with an Ar-ion beam at an incidence angle of 50°. Note that nanoripples can be induced on the initially flat surfaces of PR and ARC at an identical incidence angle of 50°, as shown in Figure 4a,b. Such nanoripples are called “intrinsic” nanoripples and are induced by IB. For the PSD curves (Figure 4d), the high-frequency peak of each PSD curve is defined as a frequency value larger than 0.005 nm^−1^, corresponding to a spatial period of 200 nm. The dominant period in each case is approximately 100 nm, corresponding to a frequency of ~0.01 nm^−1^. The intrinsic nanoripple wavelengths of the PR and ARC are close to each other with a value of ~100 nm. Moreover, the dominant periods of the bilayer cases slightly deviate from those of the intrinsic nanoripples on the single PR or ARC layer. In particular, a higher peak value around the frequency of ~0.01 nm^−1^ of the PR/ARC bilayer, together with its narrower full width at half maximum (FWHM), indicates a better lateral periodicity (ordering) of the ripples on the PR/ARC bilayer surfaces. The lateral ordering of the nanoripples on the PR/ARC bilayer is better than that on a single PR or ARC surface, which can be deduced from their PSD curves. Hence, the ordering of the nanoripples is enhanced by the bombardment of the two bilayer systems.

An intuitive understanding of the improvement in ripple ordering by IB of a bilayer system may come from the following explanation. The growth of the improved ripples in the PR/ARC bilayer systems involves three processes:

(a) The well-grown IB-induced nanoripples on the PR surface;
(b) The ripple pattern transfers from the top layer of PR to the underlying layer of ARC. This indicates that the resultant ARC ripple patterns, transferred from those in the PR layer, act as self-organized prepatterns during the sequential bombardment of the ARC surface;
(c) The evolution of the initial nonuniform ARC nanoripples into uniform ones. Considering the entire bombardment of the PR/ARC bilayer, a synergy of the pattern formation mechanisms includes pattern transfer using a sacrificial IB-induced, nanorippled mask in processes (a) and (b), and subsequent curvature-dependent sputtering based on the Bradley–Harper model [1] in process (c). In fact, the entire process rarely occurs concurrently during IB. Additionally, to achieve nanoripples with enhanced regularity, at least two conditions must be met. First, the ripple vector of the potential ripples (latent ripples on ARC) needs to be parallel to that of the existing ripples (ARC ripples transferred from the PR ripples). Second, the wavelength of the potential ripples should be close to that of the existing ripples.

## 5. Discussion

To visualize the overall picture of this topic, we summarize the various strategies for the ordering enhancement of IB-induced, self-organized nanoripple patterns in terms of sample and ion beam conditions in Table 1. In particular, based on the analysis in Section 2, Section 3 and Section 4, we see that the rocking bombardment [51,52] and the bombardment of a bilayer system [55] share the basic condition of ripple superposition. Moreover, the findings on the bombardment of the surface with grating prepatterns [54] and the initially flat surface of a bilayer system [55] provide inspiration for strategies for guided self-organization. Thus, we present our understanding of the relevant progress from the perspectives of ripple superposition and guided self-organization as follows.

### 5.1. Understanding the Ordering Improvement from the Perspective of Ripple Superimposition

The concept of ripple superposition was first theoretically proposed by G. Carter as early as 2004 [36]. One of the main challenges in this study is that the window conditions for ripple superposition are very narrow due to the simultaneous etching on the target, the so-called non-conservation of mass. Since then, despite efforts to achieve ripple superposition through theoretical and experimental approaches, the field remains in its infancy. 

The experimental results of rocking (Section 3) and traditional bombardment of PR/ARC (Section 4) have demonstrated ripple superposition. To achieve the superposition of two coherent light beams, the two beams need to have nearly the same frequency and their electric field vectors (or polarization vectors) are required to be parallel to each other [57]. Similarly, we draw an analogy between the superposition of IB-induced nanoripples and that of coherent light beams to deduce the condition of nanoripple superposition under the assumption of non-conservation of mass. This can be inferred from the common features observed in the experiments shown in Section 3.2 (rocking) and Section 4 (bilayer). For a constructive superposition of nanoripples, which results in improved ordering, the directions of the existing and latent ripples should be parallel to each other. Moreover, the wavelengths of the two sets of ripples should be as close as possible. These conditions form the fundamental basis for ripple superposition under non-conservation of mass.

Compared to the report on crossing ion beam sputtering (CIBS) [41], the first series of nanoripples were formed in the first sputtering step. The authors then rotated the sample by an azimuth angle of 90° and performed a second sputtering step. In this case, the directions of the ripple vectors in the CIBS are perpendicular to each other, which does not match the superposition condition. Therefore, the ripple superposition did not occur, as stated by the authors.

Note that the wavelength of ripples produced using the aforementioned superposition methods may slightly differ from the wavelength of ripples formed on the target surface under conventional bombardment. Therefore, the superposition of ripples offers a method of tailoring the structural parameters of ripple patterns.

Overall, recent experimental findings based on the rocking bombardment of a flat surface [52] and the traditional bombardment of a bilayer system [55] have offered viable strategies for investigating ripple ordering and ripple superposition mechanisms through various dynamic and static bombardment. All these efforts provide inspiration for basic conditions and control methods of IB to achieve ripple superposition and for feasible simulation strategies.

### 5.2. Understanding the Ordering Improvement from the Perspective of Guided Self-Organization

Using prepatterns to guide the growth of self-organized nanoripples involves the guiding strategies of self-organization during IB. In addition, guided self-organization has become a specialized research topic since the 2000s [44]. Until now, several fundamental issues in this field remain unresolved, such as the basic strategies for guided self-organization. Therefore, from an academic research perspective, the investigation of how to guide the growth of IB-induced nanoripples can provide valuable insights for the study of guided self-organization in other fields. And the investigation of the guided self-organization during IB may also enhance the ordering of nanoripples and their practical applications.

The observations presented in Section 2 and Section 4 illustrate two strategies for guiding the IB-induced nanoripples using periodic and self-organized prepatterns, respectively. The ordering of nanoripples in both cases was enhanced. These two types of prepatterns are complementary, each with its own advantages and limitations.

For the periodic prepatterns, grating templates are primarily produced using focused ion beam (FIB) etching or electron beam lithography (EBL). The critical dimensions of prepatterns reached by both technologies are close to the wavelength of the IB-induced nanoripples. Thus, both technologies are suitable for fabricating grating prepatterns for academic research on guided self-organization. For instance, grating prepatterns with optimized structural parameters (period, width, and height of ridges) can be fabricated for the investigation of the guidance mechanism (i.e., how grating prepatterns guide the growth of IB-induced nanoripples) and the quantitative relationships between the structural parameters of ripples and prepatterns for the ordering enhancement of self-organized ripples. UV interference lithography and IB, featuring the parallel writing mode, can fabricate patterns with high throughput and low cost. However, the critical dimension of patterns fabricated using UV interference lithography is larger than the wavelength of IB-induced nanoripples. As a tradeoff between pattern resolution and writing efficiency, EUV interference lithography [58] may be an alternative for fabricating periodic prepatterns.

Regarding the bombardment of the PR/ARC system in Section 4, the IB-induced nanoripples on the ARC layer act as self-organized prepatterns during IB. Such self-organized nanoripples are complementary to the periodic prepatterns fabricated by lithographic technologies. The advantage of the bilayer bombardment over others lies in the potential to integrate the fabrication of a prepattern on the upper layer and the further ripples on the underlying layer into a sequential process without involving other experimental facilities. The bilayer bombardment depends on similar IB-induced nanoripples on each single layer in a bilayer system. Therefore, future work will focus on new material combinations for bilayer or even multilayer systems to make the method applicable to a range of other substrates.

## 6. Conclusions

Ion bombardment (IB) has emerged as a promising nanofabrication tool for self-organized nanoripples. However, as an issue that has perplexed researchers for a long time, the poor ordering of IB-induced nanoripples implies academic questions about the technology, hindering its widespread application. This study reviews the few nontrivial experimental advances in the ordering enhancement of IB-induced nanoripples, aiming to uncover the underlying principles. Various strategies to enhance the quality of nanoripples are sorted up in terms of sample and ion beam conditions.

This review elucidates the connection between the strategies for improving the regularity of IB-induced nanoripples and the concept of ripple superposition raised by G. Carter in 2004 [36]. Inspired by the superposition condition of two coherent light beams, we deduce the basic condition for a constructive superposition of nanoripples, i.e., the directions of the two sets of nanoripples are parallel to each other, and the values of their wavelengths are as close as possible. Moreover, this review introduces a strategy for guiding self-organization using self-organized IB-induced ripple prepatterns, which are complementary to periodic grating prepatterns fabricated by conventional lithography. This review provides new insights for a profound understanding of ripple superposition mechanisms and facilitates practical ways to improve the ordering of nanoripples. In principle, these findings can be extended to self-organization processes in other fields, including FIB [9,10,11,12,13] and gas cluster ion beam [59,60,61,62,63,64].

Future work may extend these strategies of improving the ordering of nanoripples to other target materials based on the conditions of ripple superposition. The potential for ordering improvement by tuning the structural parameters of nanoripples can also be expected. Moreover, the complementary effects of periodic and self-organized prepatterns can be further investigated.

## Figures and Tables

**Table 1:** Summary of strategies for improving the ordering of self-organized nanoripples produced by ion bombardment.

Samples		Conventional Bombardment	Unconventional Bombardment
Material	Initial Surface		
Single material: Si	flat surface	Optimization of ion fluence [47]	Rocking [51,52], relevant to ripple superposition
			Moving [50], only theoretical study available
			Sequential ion bombardment [49]
			Intermittent [53]
	non-flat surface (i.e., prepatterned surface)	Grating-prepatterned surface [54], relevant to guided self-organization	
Bilayer systems	flat surface	Photoresist/antireflection coating [55], relevant to ripple superposition and guided self-organization	

## Supplementary Materials

The following supporting information can be downloaded at: https://www.mdpi.com/article/10.3390/nano15060438/s1, Table S1: Typical applications of IB-induced nanoripples on the surfaces of different materials. References [14,15,16,17,18,19,20,21,22,23,24,25,26,27,28,29,30,31,32] are cited in the Supplementary Materials.

## References

[1] Bradley R.M., Harper J.M.E. (1988). Theory of ripple topography induced by ion bombardment. J. Vac. Sci. Technol. A.

[2] Chan W.L., Chason E. (2007). Making waves: Kinetic processes controlling surface evolution during low energy ion sputtering. J. Appl. Phys..

[3] Frost F., Ziberi B., Schindler A., Rauschenbach B. (2008). Surface engineering with ion beams: From self-organized nanostructures to ultra-smooth surfaces. Appl. Phys. A.

[4] Muñoz-García J., Vázquez L., Castro M., Gago R., Redondo-Cubero A., Moreno-Barrado A., Cuerno R. (2014). Self-organized nanopatterning of silicon surfaces by ion beam sputtering. Mater. Sci. Eng. R Rep..

[5] Norris S.A., Aziz M.J. (2019). Ion-induced nanopatterning of silicon: Toward a predictive model. Appl. Phys. Rev..

[6] Hofsäss H., Bobes O. (2019). Prediction of ion-induced nanopattern formation using Monte Carlo simulations and comparison to experiments. Appl. Phys. Rev..

[7] Cuerno R., Kim J.S. (2020). A perspective on nanoscale pattern formation at surfaces by ion-beam irradiation. J. Appl. Phys..

[8] Vazquez L., Redondo-Cubero A., Lorenz K., Palomares F.J., Cuerno R. (2022). Surface nanopatterning by ion beam irradiation: Compositional effects. J. Phys. Condens. Matter.

[9] Lian J., Zhou W., Wei Q.M., Wang L.M., Boatner L.A., Ewing R.C. (2006). Simultaneous formation of surface ripples and metallic nanodots induced by phase decomposition and focused ion beam patterning. Appl. Phys. Lett..

[10] Wei Q., Lian J., Boatner L.A., Wang L.M., Ewing R.C. (2009). Propagation of ripples on pyrochlore induced by ion beam bombardment. Phys. Rev. B.

[11] Li P., Chen S., Dai H., Yang Z., Chen Z., Wang Y., Chen Y., Peng W., Shan W., Duan H. (2021). Recent advances in focused ion beam nanofabrication for nanostructures and devices: Fundamentals and applications. Nanoscale.

[12] Bachurin V.I., Smirnova M.A., Lobzov K.N., Lebedev M.E., Mazaletsky L.A., Pukhov D.E., Churilov A.B. (2024). Wavelike periodic structures on the Silicon surface initiated by irradiation with a focused Gallium ion beam. J. Surf. Investig. X-Ray Synchrotron Neutron Tech..

[13] Windisch M., Selmeczi D., Vida A., Dankhazi Z. (2024). Investigation of ripple formation on surface of Silicon by low-energy Gallium ion bombardment. Nanomaterials.

[14] Camellini A., Mazzanti A., Mennucci C., Martella C., Lamperti A., Molle A., Buatier de Mongeot F., Della Valle G., Zavelani-Rossi M. (2020). Evidence of plasmon enhanced charge transfer in large-area hybrid Au–MoS_2_ metasurface. Adv. Opt. Mater..

[15] Saini M., Augustine S., Ranjan M., Som T. (2020). In-plane optical anisotropy and SERS detection efficiency of self-organized gold nanoparticles on silicon nanoripples: Roles of growth angle and postgrowth annealing. Appl. Surf. Sci..

[16] Giordano M.C., Pham L.D., Ferrando G., Nguyen H.S., Le C.H., Mai T.-H., Zambito G., Gardella M., Buatier de Mongeot F. (2023). Self-organized plasmonic nanowire arrays coated with ultrathin TiO_2_ films for photoelectrochemical energy storage. ACS Appl. Nano Mater..

[17] Lamba T.K., Augustine S., Saini M., Sooraj K.P., Ranjan M. (2024). LSPR anisotropy minimization by sequential growth of Ag nanoparticles on nanoripple patterned Si surface for SERS Application. Surf. Interfaces.

[18] Kratzer M., Szajna K., Wrana D., Belza W., Krok F., Teichert C. (2018). Fabrication of ion bombardment induced rippled TiO_2_ surfaces to influence subsequent organic thin film growth. J. Phys. Condens. Matter.

[19] Giordano M.C., Sacco F.d., Barelli M., Portale G., Buatier de Mongeot F. (2021). Self-organized tailoring of faceted glass nanowrinkles for organic nanoelectronics. ACS Appl. Nano Mater..

[20] Mennucci C., Del Sorbo S., Pirotta S., Galli M., Andreani L.C., Martella C., Giordano M.C., de Mongeot F.B. (2018). Light scattering properties of self-organized nanostructured substrates for thin-film solar cells. Nanotechnology.

[21] Gupta D., Chhoker K., Rani U., Salim A., Singhal R., Sharma V., Aggarwal S. (2024). Fabrication of Ripple Structured Silicon Carbide (SiC) Films for Nano-Grating and Solar Cell Applications. ChemNanoMat.

[22] Kaur D., Rakhi, Posti R., Singh J., Roy D., Sarkar S., Kumar M. (2024). Nanopatterning Induced Si Doping in Amorphous Ga_2_O_3_ for Enhanced Electrical Properties and Ultra-Fast Photodetection. Small.

[23] Barelli M., Ferrando G., Giordano M.C., Buatier de Mongeot F. (2022). Wavelength-Dependent Plasmonic Photobleaching of Dye Molecules by Large-Area Au Nanostripe Arrays. ACS Appl. Nano Mater..

[24] Arranz M.A., Colino J.M., Palomares F.J. (2014). On the limits of uniaxial magnetic anisotropy tuning by a ripple surface pattern. J. Appl. Phys..

[25] Bera A.K., Dev A.S., Kumar D. (2023). Enhancing the limit of uniaxial magnetic anisotropy induced by ion beam erosion. Appl. Phys. Lett..

[26] Yang Y., Keller A. (2021). Ion beam nanopatterning of biomaterial surfaces. Appl. Sci..

[27] Garcia M.A., Gago R., Arroyo-Hernández M., de Laorden E.H., Iglesias M., Esteban-Mendoza D., Cuerno R., Rickards J. (2023). Texturization of polycrystalline titanium surfaces after low-energy ion-beam irradiation: Impact on biocompatibility. Surf. Coat. Technol..

[28] Pachchigar V., Parida B.K., Augustine S., Hans S., Saini M., Sooraj K.P., Ranjan M. (2023). Comparative wettability study of bulk and thin film of polytetrafluoroethylene after low energy ion irradiation. Thin Solid Film..

[29] Vandana, Kumar T., Ojha S., Kumar S. (2021). Energy-dependent surface nanopatterning of Si (100) for different projectiles: A tunable anisotropic wettability of ripple surface. Appl. Nanosci..

[30] Hans S., Parida B.K., Augustine S., Pachchigar V., Sooraj K.P., Ranjan M. (2024). Anisotropic wettability transition on nanoterraced glass surface by Ar ions. J. Mater. Sci..

[31] Jacak W.A. (2020). Quantum Nano-Plasmonics.

[32] Luo P., Jaramillo C., Wallum A.M., Liu Z., Zhao R., Shen L., Zhai Y., Spear J.C., Curreli D., Lyding J.W. (2020). Coherent Atomic-Scale Ripples on Metallic Glasses Patterned by Low-Energy Ion Irradiation for Large-Area Surface Structuring. ACS Appl. Nano Mater..

[33] Buatier de Mongeot F., Valbusa U. (2009). Applications of metal surfaces nanostructured by ion beam sputtering. J. Phys. Condens. Matter.

[34] Seo J., Pearson D.A., Bradley R.M., Kim J.S. (2022). Nanoscale pattern formation on silicon surfaces bombarded with a krypton ion beam: Experiments and simulations. J. Phys. Condens. Matter.

[35] Rüdiger T., Mitzschke M., Bundesmann C., Prager A., Liu Y., Abel B., Schulze A., Frost F. (2024). Ion incidence angle-dependent pattern formation on AZ^®^ 4562 photoresist by reactive ion beam etching. Surf. Coat. Technol..

[36] Carter G. (2004). Proposals for producing novel periodic structures by ion bombardment sputtering. Vacuum.

[37] Carter G. (2005). Surface ripple amplification and attenuation by sputtering with diametrically opposed ion fluxes. Vacuum.

[38] Vogel S., Linz S.J. (2007). Surface structuring by multiple ion beams. Phys. Rev. B.

[39] Joe M., Choi C., Kahng B., Kim J.-S. (2007). Nanopatterning by dual-ion-beam sputtering. Appl. Phys. Lett..

[40] Joe M., Kim J.H., Choi C., Kahng B., Kim J.S. (2009). Nanopatterning by multiple-ion-beam sputtering. J. Phys. Condens. Matter.

[41] Kim J.H., Joe M., Kim S.P., Ha N.B., Lee K.R., Kahng B., Kim J.S. (2009). Pattern evolution on previously rippled Au(001) by crossing-ion-beam sputtering. Phys. Rev. B.

[42] Kim J.H., Kim J.-S., Muñoz-García J., Cuerno R. (2013). Role of nonlinearities and initial prepatterned surfaces in nanobead formation by ion-beam bombardment of Au(001): Experiments and theory. Phys. Rev. B.

[43] Muñoz-García J., Cuerno R., Castro M. (2008). Coupling of morphology to surface transport in ion-beam irradiated surfaces: Oblique incidence. Phys. Rev. B.

[44] Prokopenko M. (2009). Guided self-organization. HFSP J..

[45] Araujo N.A.M., Janssen L.M.C., Barois T., Boffetta G., Cohen I., Corbetta A., Dauchot O., Dijkstra M., Durham W.M., Dussutour A. (2023). Steering self-organisation through confinement. Soft Matter.

[46] Li H., Li J., Yang G., Liu Y., Frost F., Hong Y. (2023). Can one series of self-organized nanoripples guide another series of self-organized nanoripples during ion bombardment: From the perspective of power spectral density entropy?. Entropy.

[47] Keller A., Facsko S., Möller W. (2008). Minimization of topological defects in ion-induced ripple patterns on silicon. New J. Phys..

[48] Ziberi B., Cornejo M., Frost F., Rauschenbach B. (2009). Highly ordered nanopatterns on Ge and Si surfaces by ion beam sputtering. J. Phys. Condens. Matter.

[49] Keller A., Facsko S. (2010). Tuning the quality of nanoscale ripple patterns by sequential ion-beam sputtering. Phys. Rev. B.

[50] Gelfand M.P., Bradley R.M. (2012). Highly ordered nanoscale patterns produced by masked ion bombardment of a moving solid surface. Phys. Rev. B.

[51] Harrison M.P., Bradley R.M. (2016). Producing virtually defect-free nanoscale ripples by ion bombardment of rocked solid surfaces. Phys. Rev. E.

[52] Jo S., Jun J., Lee E., Yoon S.M., Seo J., Muñoz-García J., Cuerno R., Kim J.S. (2020). Order improvement of surface nanopatterns via substrate rocking under ion bombardment: Experiments and nonlinear models. Phys. Rev. B.

[53] Rakhi, Muñoz-García J., Cuerno R., Sarkar S. (2023). Towards ordered Si surface nanostructuring: Role of an intermittent ion beam irradiation approach. Phys. Scr..

[54] Cuenat A., George H.B., Chang K.C., Blakely J.M., Aziz M.J. (2005). Lateral templating for guided self-organization of sputter morphologies. Adv. Mater..

[55] Li J., Yang G., Bradley R.M., Liu Y., Frost F., Hong Y. (2021). Enhancing the quality of self-organized nanoripples by Ar-ion bombardment of a bilayer system. Nanotechnology.

[56] Cuerno R., Barabási A.-L. (1995). Dynamic scaling of ion-sputtered surfaces. Phys. Rev. Lett..

[57] Hecht E., Zajac A. (1974). Optics.

[58] Kazazis D., Santaclara J.G., von Schoot J., Mochi I., Ekinci Y. (2024). Extreme ultraviolet lithography. Nat. Rev. Methods Primers.

[59] Yamada I. (2001). Materials processing by gas cluster ion beams. Mater. Sci. Eng. R Rep..

[60] Tilakaratne B., Chen Q., Chu W.-K. (2017). Self-Assembled Gold Nano-Ripple Formation by Gas Cluster Ion Beam Bombardment. Materials.

[61] Toyoda N., Tilakaratne B., Saleem I., Chu W.-K. (2019). Cluster beams, nano-ripples, and bio applications. Appl. Phys. Rev..

[62] Pelenovich V., Zeng X., Rakhimov R., Zuo W., Tian C., Fu D., Yang B. (2020). Decoration of ZnO needles with nanoripples using gas cluster ion bombardment. Mater. Lett..

[63] Ieshkin A., Kireev D., Ozerova K., Senatulin B. (2020). Surface ripples induced by gas cluster ion beam on copper surface at elevated temperatures. Mater. Lett..

[64] Jiménez-Sáez J.C., Muñoz S., Palacios P. (2024). Surface Ripple Formation by Bombardment with Clusters: Influence of Mass. Appl. Sci..

