# Supplementary material for: Ordering Enhancement of Ion Bombardment-Induced Nanoripple Patterns: A Review
Source: Nanomaterials (Basel). 2025 Mar 13;15(6):438. doi: 10.3390/nano15060438 (PMC11944364; doi:10.3390/nano15060438)
Supplement: Supplementary file 1 [file nanomaterials-15-00438-s001.zip › nanomaterials-3451864-supplementary.pdf]

Supplementary Materials

Ordering Enhancement of Ion Bombardment-Induced Nanoripple Patterns: A Review

Ying Liu [1,*], Hengbo Li [1], Chongyu Wang [1], Gaoyuan Yang [1], Frank Frost [2] and Yilin Hong [1]

[1] National Synchrotron Radiation Laboratory, University of Science and Technology of China, Hezuohua South Road 42, Hefei 230029, China; lihengbo@mail.ustc.edu.cn (H.L.); sa24231065@mail.ustc.edu.cn (C.W.); ygyuan@mail.ustc.edu.cn (G.Y.); ylhong@ustc.edu.cn (Y.H.)
[2] Leibniz Institute of Surface Engineering (IOM), Permoserstraße 15, 04318 Leipzig, Germany; frank.frost@iom-leipzig.de
* Correspondence: liuychch@ustc.edu.cn

Table S1 provides detailed the information of IB-induced nanoripples and main bombardment conditions of different applications. The fields of these applications span multiple fields, inculding surface plasmonics [1–4], optoelectronics [5,6], photovoltaics [7,8], flexible electronics [9], photocatalysis [10], magnetism [11,12], biomaterials [13,14], and wettability [15–17]. Moreover, the nanoripples can be formed on a wide range of solid surfaces such as metals, semiconductors, insulators and polymers. The structural parameters of nanoripples depend on target materials and ion bombardment conditions. For example, the periods of nanoripples can vary from 20 nm to 300 nm – 400 nm. Normally noble gases like Ar and Kr, with ion energy ranging from 300 eV to 100 keV can be used for bombardment. Note that the actual characterization of IB-induced nanoripples for quantum nanoplasmonics [18,19] is not available. Nevertheless, the IB-induced nanostructures including nanoripples can be used in quantum nanoplasmonics [18,19].

References

1. Camellini, A.; Mazzanti, A.; Mennucci, C.; Martella, C.; Lamperti, A.; Molle, A.; Buatier de Mongeot, F.; Della Valle, G.; Zavelani-Rossi, M. Evidence of plasmon enhanced charge transfer in large-area hybrid Au–MoS_2 metasurface. *Advanced Optical Materials* **2020**, *8*, 2000653, doi:10.1002/adom.202000653.
2. Saini, M.; Augustine, S.; Ranjan, M.; Som, T. In-plane optical anisotropy and SERS detection efficiency of self-organized gold nanoparticles on

silicon nanoripples: Roles of growth angle and postgrowth annealing. *Applied Surface Science* **2020**, *512*, 145703, doi:10.1016/j.apsusc.2020.145703.

3. Giordano, M.C.; Pham, L.D.; Ferrando, G.; Nguyen, H.S.; Le, C.H.; Mai, T.-H.; Zambito, G.; Gardella, M.; Buatier de Mongeot, F. Self-organized plasmonic nanowire arrays coated with ultrathin TiO_2 films for photoelectrochemical energy storage. *ACS Applied Nano Materials* **2023**, *6*, 21579-21586, doi:10.1021/acsanm.3c03546.
4. Lamba, T.K.; Augustine, S.; Saini, M.; Sooraj, K.P.; Ranjan, M. LSPR anisotropy minimization by sequential growth of Ag nanoparticles on nanoripple patterned Si surface for SERS Application. *Surfaces and Interfaces* **2024**, *52*, 104852, doi:10.1016/j.surfin.2024.104852.
5. Kratzer, M.; Szajna, K.; Wrana, D.; Belza, W.; Krok, F.; Teichert, C. Fabrication of ion bombardment induced rippled TiO_2 surfaces to influence subsequent organic thin film growth. *Journal of Physics: Condensed Matter* **2018**, *30*, 283001, doi:10.1088/1361-648X/aac758.
6. Giordano, M.C.; Sacco, F.d.; Barelli, M.; Portale, G.; Buatier de Mongeot, F. Self-organized tailoring of faceted glass nanowrinkles for organic nanoelectronics. *ACS Applied Nano Materials* **2021**, *4*, 1940-1950, doi:10.1021/acsanm.0c03290.
7. Mennucci, C.; Del Sorbo, S.; Pirotta, S.; Galli, M.; Andreani, L. C.; Martella, C.; Giordano, M.C.; de Mongeot, F.B. Light scattering

properties of self-organized nanostructured substrates for thin-film solar cells. *Nanotechnology* **2018**, *29*, 355301, doi: 10.1088/1361-6528/aac9ac.

8. Gupta, D.; Chhoker, K.; Rani, U.; Salim, A.; Singhal, R.; Sharma, V.; Aggarwal, S. Fabrication of Ripple Structured Silicon Carbide (SiC) Films for Nano-Grating and Solar Cell Applications. *ChemNanoMat* **2024**, *11*, 202400455, doi:10.1002/cnma.202400455.

9. Kaur, D.; Rakhi; Posti, R.; Singh, J.; Roy, D.; Sarkar, S.; Kumar, M. Nanopatterning Induced Si Doping in Amorphous Ga_2O_3 for Enhanced Electrical Properties and Ultra-Fast Photodetection. *Small* **2024**, *20*, 2309277, doi:10.1002/smll.202309277.

10. Barelli, M.; Ferrando, G.; Giordano, M.C.; Buatier de Mongeot, F. Wavelength-Dependent Plasmonic Photobleaching of Dye Molecules by Large-Area Au Nanostripe Arrays. *ACS Applied Nano Materials* **2022**, *5*, 3470-3479, doi:10.1021/acsanm.1c04087.

11. Arranz, M.A.; Colino, J.M.; Palomares, F.J. On the limits of uniaxial magnetic anisotropy tuning by a ripple surface pattern. *Journal of Applied Physics* **2014**, *115*, 183906, doi:10.1063/1.4876232.

12. Bera, A.K.; Dev, A.S.; Kumar, D. Enhancing the limit of uniaxial magnetic anisotropy induced by ion beam erosion. *Applied Physics Letters* **2023**, *122*, 022405, doi:10.1063/5.0125851.

13. Yang, Y.; Keller, A. Ion beam nanopatterning of biomaterial surfaces.

Applied Sciences **2021**, *11*, 6575, doi:10.3390/app11146575.

14. Garcia, M.A.; Gago, R.; Arroyo-Hernández, M.; de Laorden, E.H.; Iglesias, M.; Esteban-Mendoza, D.; Cuerno, R.; Rickards, J. Texturization of polycrystalline titanium surfaces after low-energy ion-beam irradiation: Impact on biocompatibility. *Surface and Coatings Technology* **2023**, *458*, 129363, doi:10.1016/j.surfcoat.2023.129363.
15. Pachchigar, V.; Parida, B.K.; Augustine, S.; Hans, S.; Saini, M.; Sooraj, K.P.; Ranjan, M. Comparative wettability study of bulk and thin film of polytetrafluoroethylene after low energy ion irradiation. *Thin Solid Films* **2023**, *777*, 139888, doi:10.1016/j.tsf.2023.139888.
16. Vandana; Kumar, T.; Ojha, S.; Kumar, S. Energy-dependent surface nanopatterning of Si (100) for different projectiles: a tunable anisotropic wettability of ripple surface. *Applied Nanoscience* **2021**, *13*, 3189-3196, doi:10.1007/s13204-021-01975-5.
17. Hans, S.; Parida, B.K.; Augustine, S.; Pachchigar, V.; Sooraj, K.P.; Ranjan, M. Anisotropic wettability transition on nanoterraced glass surface by Ar ions. *Journal of Materials Science* **2024**, *59*, 14205-14223, doi:10.1007/s10853-024-10039-2.
18. WITOLD A. JACAK. QUANTUM NANO-PLASMONICS; Cambridge University Press, 2020; pp. 1-16,
19. Luo, P.; Jaramillo, C.; Wallum, A.M.; Liu, Z.; Zhao, R.; Shen, L.; Zhai, Y.; Spear, J.C.; Curreli, D.; Lyding, J.W.; et al. Coherent Atomic-Scale

Ripples on Metallic Glasses Patterned by Low-Energy Ion Irradiation for Large-Area Surface Structuring. *ACS Applied Nano Materials* **2020**, *3*, 12025-12033, doi:10.1021/acsanm.0c02548.

Table S1. Typical applications of IB-induced nanoripples on the surfaces of different materials

Application	Ripple material	Structural parameters of ripples (period, rms roughness or height) /nm	Ion species	Ion energy / eV	Ion fluence / ions/cm^2	Ref
Plasmonics	Au	(~80, /)	Ar^+	800	1.2×10^{18}	1
Plasmonics	Si	(30, 3)	Ar^+	500	5×10^{17}	2
Plasmonics	Glass	(260, 80–100)	Ar^+	800	/	3
Plasmonics	Si	(30, 2)	Ar^+	500	3×10^{18}	4
Opto-electronics	TiO_2	(30, 1.9)	Ar^+	2000	1.9×10^{19}	5
Opto-electronics	Glass	(200, 60–70)	Ar^+	800	1.4×10^{19}	6
Photovoltaics	Au	(~80, /)	Ar^+	800	1.2×10^{18}	7
Photovoltaics	SiC	(370, 18)	Ar^+	20k	8×10^{17}	8
Flexible electronics	SiO_2	(39, 1)	Ar^+	500	/	9
Photocatalysis	Glass	(140, 30)	Ar^+	800	2.8×10^{19}	10
Magnetism	Co	(60–75, 5–10.8)/	Ar^+	1200	0.63×10^{14}	11
Magnetism	Co	(650, 1.17)	/	/	/	12
Biomaterials	Various solid materials	(20–several hundred, /)	/	/	/	13
Biomaterials	Polycrystalline Ti	(~30, /)	Ar^+	1k	1×10^{18}	14
Wettability	polytetrafluoroethylene	(~65–76, /) (~128–212, /)	Ar^+	300, 800	/	15
Wettability	Si	(~700–900, /) (~450–650, /)	Ar^+, Kr^+	60k, 80k, 100k	7×10^{17}	16
Wettability	Glass	(53, 4) (~90, 30–40)	Ar^+	1000	9.7×10^{17} 6.9×10^{18}	17
